# EthnoHERBS: Harnessing traditional herbal knowledge for biodiversity conservation and innovative health solutions

**DOI:** 10.1016/j.csbj.2025.03.035

**Published:** 2025-03-25

**Authors:** Dimitrios Zouraris, Konstantia Graikou, Panagiotis Vasileiou, Vladimir Dimitrov, Zora Dajic Stevanovic, Anna Rita Bilia, Jelena Zivkovic, Alberto Dias, Konstantinos Kasiotis, Konstantinos Gardikis, Paula Dias, Mirko Oluški, Juan Ramón Muñoz Montaño, Hristina Hristova, Hristo Iliev, Giovanna Petrangolini, Antreas Afantitis, Nektarios Aligiannis

**Affiliations:** a NovaMechanics Ltd, Nicosia 1070, Cyprus; b Entelos Institute, Larnaca 6059, Cyprus; c Department of Pharmacognosy and Natural Products Chemistry, Faculty of Pharmacy, National and Kapodistrian University of Athens, Panepistimiopolis Zografou, Athens 15771, Greece; d Institute of Organic Chemistry with Centre of Phytochemistry, Bulgarian Academy of Sciences, Acad. Georgi Bonchev 9, Sofia 1113, Bulgaria; e Faculty of Agriculture, University of Belgrade, Nemanjina 6, Belgrade 11080, Serbia; f Department of Chemistry “Ugo Schiff”, University of Florence, Piazza San Marco 4, Florence 50121, Italy; g Institute for Medicinal Plants Research “Dr Josif Pančić”, Tadeuša Košćuška 1, Belgrade 171000, Serbia; h Department of Biology and CITAB-UM research Center, University of Minho, Largo do Paço, Braga 4704-553, Portugal; i Benaki Phytopathological Institute, Stefanou Delta street 8, Athens 14561, Greece; j Apivita SA, Industrial Park of Markopoulo, Athens 19003, Greece; k TecMinho, Universidade do Minho, Campus de Azurém, Guimarães 4800-058, Portugal; l Euro Herbs d.o.o., Lovćenska 7, Novi Sad 21000, Serbia; m Zurko Research SL, Calle Gran via 62, 4 Izq, Madrid 28013, Spain; n VenusRoses Labsolutions Ltd., Department of Research and Innovation, 111, Tsarigradsko shose blvd, Sofia 1784, Bulgaria; o GALEN-N Ltd., 23, Tvardishki Prohod Str., Sofia 1404, Bulgaria; p Indena S.p.A., Via Don Minzoni 6, Settala 20049, Italy; q NovaMechanics MIKE, Piraeus 18545, Greece

**Keywords:** Ethnobotany, Traditional herbal knowledge, Natural product chemistry, Biodiversity conservation, Cosmeceutical Innovation

## Abstract

EthnoHERBS represents a pioneering multidisciplinary initiative that integrates traditional herbal knowledge with advanced natural product chemistry to promote biodiversity conservation and foster innovative cosmeceutical solutions. The project systematically documents centuries-old ethnobotanical practices across South-Eastern Europe, leading to the identification of a diverse array of medicinal and aromatic plants traditionally used to treat skin disorders. Employing environmentally friendly extraction techniques alongside cutting-edge analytical tools—including UHPLC-HRMS, CPC, and NMR spectroscopy—over 500 bioactive compounds have been characterized, with 30 novel secondary metabolites isolated and structurally elucidated. Advanced *in silico* methodologies, such as docking, molecular dynamics simulations, and MM-GBSA rescoring, were implemented to evaluate the interaction profiles of these compounds with key skin disorder-related enzymes, including elastase, tyrosinase, hyaluronidase, and xanthine oxidase. Complementary in vitro and in vivo assays confirmed the potent antioxidant, anti-inflammatory, and wound-healing properties of the selected extracts. Furthermore, the project underscores sustainable practices by establishing organic cultivation protocols and pilot-scale production processes, ensuring the eco-friendly exploitation of natural resources. By fostering extensive collaboration between academic institutions and industry partners under the Horizon 2020 framework, EthnoHERBS not only advances scientific research and innovative product development but also serves as a model for preserving traditional knowledge and biodiversity.

## Graphical Abstract

**Figure:**
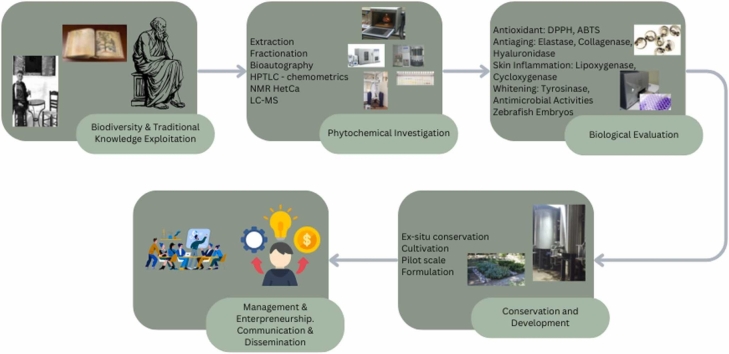

## 1 Introduction

The World Health Organisation (WHO), through “WHO Traditional Medicine Strategies 2014–2023,” has been promoting the idea that ethnobotanical information can lead to valuable drug discovery. Proof of this concept is exemplified by the Nobel Prize in Physiology and Medicine, awarded in 2015 to Prof. Youyou Tu for the discovery of artemisinin—a secondary metabolite of the plant species Artemisia annua. This compound is used as a potent antimalarial agent against Plasmodium strains resistant to all known antimalarials. Prof. Youyou Tu and co-workers studied over 1200 recipes from Chinese Traditional Medicine, ultimately formulating the preparation known as “Quinhosu,” which proved ideal for treating chills and fever. One of the major advantages of choosing plants as the starting point in drug development through ethnobotanical surveys is that their active constituents—having undergone long-term use by mankind—are likely to be safer than those isolated from plants without a history of ethnomedical use. This long-term usage provides additional confidence in their safety. Despite its critical importance, biodiversity is under threat from various human activities, including habitat destruction, overexploitation of resources, pollution, climate change, and the introduction of invasive species. These factors are major drivers of biodiversity loss [1] eventually leading to ecosystem degradation, species loss, and a decline in genetic diversity—with profound implications for ecosystem services and human livelihoods[2].

Traditional herbal knowledge, accumulated over centuries by indigenous and local communities, represents a valuable repository of information about the medicinal and aromatic properties of plants encapsulating the identification, use, and management of plant species for health and well-being [3] while plays a crucial role in biodiversity conservation by promoting sustainable harvesting practices, the protection of plant habitats, and the cultivation of medicinal plants [4]. According to WHO, more than 80 % of the world’s population still relies on herbal traditional medicines for healing a variety of diseases, including several skin disorders. Traditional medicine encompasses the knowledge, skills, and practices based on the theories, beliefs, and experiences of indigenous populations from different cultures. This knowledge continues to inspire the search for new therapeutic agents, particularly in the face of emerging health challenges and the need for alternative treatments [5], [6]. As in medicine, traditional herbal knowledge has provided the foundation for numerous modern pharmaceuticals—many widely used drugs, such as aspirin, quinine, and artemisinin, have been derived from plants used in traditional medicine. In the ancient Western world, the Greeks contributed significantly to the rational development of herbal drugs. Although the healing properties of plants were known since the time of Homer, it was the work of Hippocrates (5th century BC) and Dioscorides (1st century AD) that established medicine as a science rooted in the therapeutic properties of plants. During the Middle Ages, monks preserved and disseminated this knowledge by writing textbooks on medicinal plants, thereby safeguarding the ancient Greek medical tradition. Notably, in the 17th, 18th, and 19th centuries, “Zagori” emerged as a renowned centre of traditional medicine in the Balkan Peninsula. However, after the 1960s, there was a significant decline in the use of medicinal plants, and while traditional healing approaches from China and India have seen a revival in Europe in recent decades, the roots of European traditional medicine have gradually faded.

Traditional knowledge and modern science offer complementary perspectives that, when integrated, can enhance our understanding and management of biodiversity. They also provide insights into the ecological and cultural context of plant use, offering practical knowledge about species’ distribution, growth habits, and therapeutic properties [7]. This traditional knowledge is often based on long-term observations and experiences, which can inform sustainable practices and local conservation effort [8]. Modern science, on the other hand, brings systematic methodologies, advanced technologies, and a theoretical framework to the study of biodiversity. Scientific research can validate and expand traditional knowledge, providing empirical evidence for the efficacy of herbal remedies and uncovering the biochemical mechanisms underlying their therapeutic effects [9]. Modern techniques in phytochemistry, molecular biology, and ecological modelling can enhance the identification, characterization, and conservation of plant species [10]. The integration of these knowledge systems can lead to innovative approaches to biodiversity conservation and the development of new therapeutic products. Collaborative research that respects and incorporates traditional knowledge can generate holistic solutions that are scientifically robust and culturally appropriate [7].

The Balkans is a region characterised by exceptionally high biodiversity and endemism, yet it faces significant challenges stemming from deforestation, agricultural expansion, urbanisation, and industrial activities [11]. Political and economic transitions have further contributed to inadequate environmental governance and conservation efforts, exacerbating pressures on. Additionally, the loss of traditional land-use practices—which historically maintained landscape and habitat diversity—continues to threaten the region’s ecological integrity [12]. Against this backdrop, the Balkans has a long history of employing medicinal and aromatic plants for traditional healing, dating back to ancient times, with a notable modern revival of this practice [13]. The systematic collection of information on the traditional uses of herbs in South-Eastern European countries has been a key objective of the EthnoHERBS project. This collection process has involved searches in digital databases containing ancient texts, manuscript archives, and historical records of the Balkan territory, as well as the use of questionnaires, semi-structured interviews, informal interviews, and discussions with elders and physicians during ethnobotanical surveys. A great number of these plant species exhibit healing potential and are used to treat diverse skin diseases. Over the last decade, the region has become a focal point of numerous ethnobotanical studies highlighting its rich biodiversity [14], [15]. A study in the Zlatibor district (South-western Serbia) found that 29.6 % of traditionally used species target dermatological conditions [16]. Although many plants have shown excellent results, an insufficient number of clinical studies has been conducted to elucidate their actual therapeutic effects [17], [18]. Across Central and Western Balkans, large numbers of medicinal plant species are utilised, reflecting the region’s remarkable plant biodiversity [14], [15], [19]. For example, in Serbia around 700 species (approximately 10.7 % of total plant taxa) are employed, whereas in Croatia up to 21 % of its total 5000 taxa find similar usage [14]. Many Balkan species are traditionally administered to manage a broad spectrum of dermatological conditions [20], notably eczema [21]. Although extensive ethnobotanical knowledge indicates promising results for numerous plant remedies, comparatively few clinical studies have been conducted to substantiate and clarify their actual effects [17].

The EthnoHERBS project (full title *“Conservation of European Biodiversity through Exploitation of Traditional Herbal Knowledge for the Development of Innovative Products”*) is an ambitious research initiative that harnesses the rich ethnobotanical knowledge and biodiversity of the Balkan Peninsula. As a H2020-MSCA-RISE project, EthnoHERBS aims to record and evaluate information on South-Eastern (SE) European traditional knowledge, explore the region’s flora in a high-throughput manner, and utilise cutting-edge technologies in Natural Products Chemistry to discover and develop innovative cosmeceutical products against skin disorders. At its core, the project relies on the systematic collection and analysis of ethnobotanical data, coupled with advanced chemical and biological methodologies to extract, purify, and characterise bioactive compounds from the selected plants. A total of 198 ethnobotanical studies have been identified across the Balkans and South-Eastern European countries, yielding a wealth of data on traditional medicinal practices [22]. Additionally, a separate review focusing on the treatment of skin disorders in Albania, Cyprus, Greece, and Turkey identified 967 taxa, belonging to 418 different genera and 111 different families. This review demonstrated the significant breadth of plant usage for skin-related conditions in the region, and it found that the most commonly used species include *Plantago major L.*, *Hypericum perforatum L.*, and *Sambucus nigra L.* [22].

On of the main concerns of the project is sustainability as it emphasises on the organic cultivation of selected plants, aiming to preserve biodiversity whilst promoting the sustainable exploitation of natural resources. After having integrated advanced scientific techniques with traditional herbal knowledge, the consortium aspires to ultimately produce high-quality herbal extracts and bioactive compounds—particularly those relevant to skin disorders. To do so, the project aims to optimise production processes and harness eco-friendly technologies for extraction and formulation. Moreover, one key element of the EthnoHERBS project is that it is based on extensive collaboration comprising seven academic institutions and seven non-academic partners from five EU Member States and one Candidate Country, the consortium is structured to maximise knowledge exchange through a robust secondments scheme. While, academic partners bring expertise in ethnobotany, phytochemistry, and biology, small- and medium-sized enterprises (SMEs) contribute practical insights for commercialising plant-derived products so that ultimately, EthnoHERBS can establish a successful model of international collaboration that will serve as a benchmark for future endeavours aimed at conserving biodiversity and sustainably utilising natural resources. This approach highlights the value of scientific excellence, sustainable practices, and smooth knowledge transfer from research to market application. By doing so, the project’s outcomes are poised to generate lasting benefits not only for the scientific community but also for society as a whole.

## 2 Project description

### 2.1 Scope and objectives

The scope of the EthnoHERBS project encompasses the systematic exploration and utilization of medicinal and aromatic plants from the Balkan Peninsula. It aims to integrate traditional ethnobotanical knowledge with advanced scientific techniques in Natural Product Chemistry. The project spans a comprehensive workflow from the collection and classification of traditional herbal knowledge to the development and commercialization of innovative products derived from these plants. The project is centred around the conservation and sustainable use of biodiversity. This involves conserving plant biodiversity through the organic cultivation of selected medicinal and aromatic plants. Ensuring the sustainable exploitation of these plants is achieved by adhering to eco-friendly extraction and processing methods, which minimize environmental impact and promote sustainability. Another key objective of the project is the integration of traditional knowledge with modern science. This entails collecting, documenting, and analysing traditional herbal knowledge from the Balkan Peninsula. By applying this traditional knowledge in conjunction with advanced techniques in Natural Product Chemistry, the project aims to discover bioactive compounds that can be used in innovative therapeutic applications. The development of innovative products is a critical component of the EthnoHERBS project. The focus is on developing high-quality herbal extracts and compounds with therapeutic potential, particularly for treating skin disorders. The project also seeks to optimize production processes and formulation methods to create products that are ready for market introduction, ensuring their efficacy, safety, and stability. Furthermore, the project promotes international and intersectoral collaboration. This is fostered through an extensive secondments scheme that encourages collaboration between academic institutions and non-academic partners. By facilitating the exchange of knowledge and best practices among experts in various fields, the EthnoHERBS project aims to enhance its scientific and practical outcomes, ensuring a broad and lasting impact on the conservation of biodiversity and the development of innovative herbal products.

### 2.2 Methodologies

**Fig. 1:**
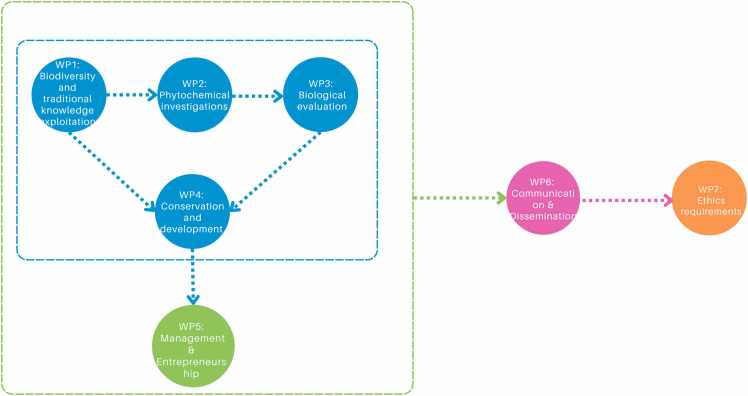
EthnoHERBS WP Interaction.

The EthnoHERBS project employs a comprehensive and systematic approach, utilizing a variety of methodologies across different work packages to achieve its objectives effectively as depicted in Fig. 1. The first step involves conducting ethnobotanical surveys and data collection. This includes conducting interviews with key informants to gather traditional knowledge about the use of herbs for treating skin disorders, as well as collecting data from ancient texts, scientific publications, and previous ethnobotanical surveys (WP1). Once the data is collected, it undergoes a process of classification and prioritization. This involves evaluating and classifying the information based on ethnobotanical principles, scientific data, and *in silico* screening. The herbs are then prioritized according to their relevance and potential therapeutic benefits, ensuring the focus is on the most promising candidates. Phytochemical investigations form the next phase of the project. This involves developing and profiling herbal preparations using environmentally friendly extraction techniques, such as Ultrasound Assisted Extraction. Advanced methodologies like chemometrics and heterocovariance are implemented for the efficient identification of bioactive components. Additionally, metabolomics and dereplication approaches are applied to avoid the isolation of already known compounds, ensuring the discovery of novel bioactives (WP2). Meanwhile, biological evaluation is a critical component of the project, where the pharmacological properties of herbal extracts, fractions, and pure compounds are assessed through a series of *in vitro* and *in vivo* assays. This includes evaluating the antioxidant, anti-inflammatory, and wound-healing properties of these compounds using various cell-free assays, cell-based assays, and zebrafish models (WP3).

Then, the conservation and development phase involves propagating and cultivating selected plants using organic methods to ensure high-quality plant material. This is followed by pilot-scale production of extracts and pure compounds, optimizing the extraction and isolation processes to ensure scalability for industrial applications. The final products are formulated and subjected to stability tests to confirm their efficacy and shelf-life (WP4). Effective management and entrepreneurship are also crucial for the success of the project. This includes coordinating all project activities and ensuring seamless collaboration among partners. Training is provided on entrepreneurship, intellectual property management, commercialization, and regulatory affairs to enhance the project's impact and ensure the successful translation of research findings into marketable products (WP5). At the same time, communication and dissemination play a vital role in the project's outreach efforts. This involves promoting the project through a well-designed outreach plan that includes constructing a project website, maintaining a social media presence, and organizing workshops and final events. Research findings are disseminated to the scientific community through publications in peer-reviewed journals and presentations at international conferences, ensuring widespread awareness and engagement with the project's outcomes (WP6).

### 2.3 Consortium description & funding

**Fig. 2:**
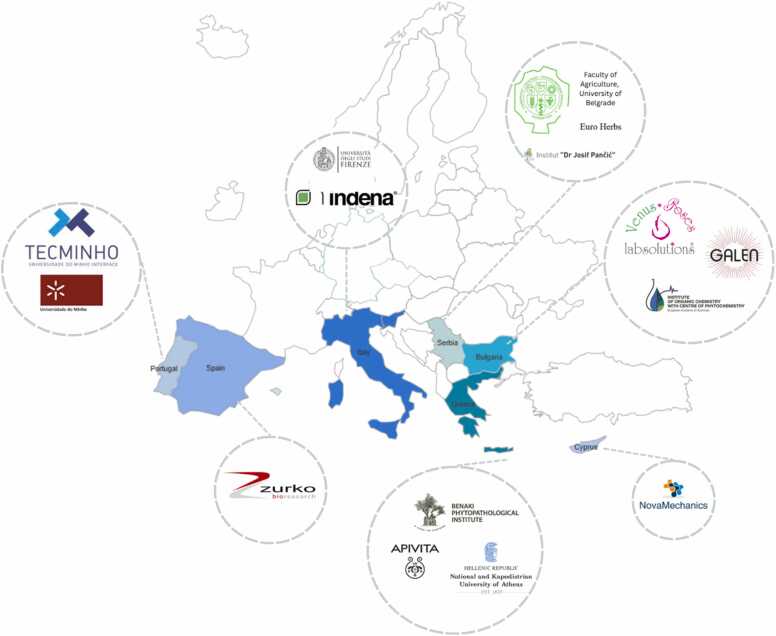
EthnoHERBS Consortium.

The EthnoHERBS project boasts a diverse and interdisciplinary consortium comprising both academic and non-academic partners, each contributing their unique expertise to achieve the project's objectives (Fig. 2). The National and Kapodistrian University of Athens in Greece coordinates the project, overseeing its activities and facilitating strong collaboration among all participants. The Faculty of Agriculture at the University of Belgrade in Serbia leads research efforts related to biodiversity. The Institute of Organic Chemistry with Centre of Phytochemistry at the Bulgarian Academy of Sciences in Bulgaria specializes in phytochemical research. The University of Florence in Italy supports formulation methods and the development of end products. The Universidade do Minho in Portugal handles the biological testing of botanical extracts and related compounds. The Benaki Phytopathological Institute in Greece engages in research and development initiatives while the Dr. Josif Pancic Institute for the Study of Medicinal Herbs in Serbia contributes to data gathering and plant propagation. Zurko Research SL in Spain advises on regulatory matters and commercialization strategies. Indena SPA in Italy carries out pilot-scale extraction and isolation processes. APIVITA company in Greece manages the conservation and development tasks, with a focus on organic cultivation and the formulation of new products. Novamechanics Limited in Cyprus lends expertise in computer-based screening and modeling. TecMinho in Portugal provides training services in entrepreneurship and intellectual property management. Venus Solutions in Bulgaria supports multiple stages of product development and evaluation and EuroHerbs DOO in Serbia emphasizes the sustainable cultivation and utilization of medicinal plants, while GALEN in Bulgaria aids in plant cultivation and pilot-scale production. The EthnoHERBS project is primarily funded through the European Union’s Horizon 2020 program under the Marie Skłodowska-Curie Actions (MSCA) Research and Innovation Staff Exchange (RISE) scheme. The project has been allocated significant financial resources to support its extensive research activities, collaboration efforts, and dissemination plans over its 66-month duration. The Horizon 2020 funding facilitates the cross-border and cross-sectoral exchange of knowledge and expertise, ensuring the project's objectives are met effectively and efficiently.

## 3 Impact

### 3.1 Biodiversity and traditional knowledge exploitation

During the project’s initial year, efforts cantered on documenting traditional plant-based remedies for skin disorders in South-Eastern Europe. This process involved consulting an array of historical and contemporary sources, encompassing ancient and Byzantine manuscripts, published ethnobotanical research, and direct interviews with local communities. From a total of 198 ethnobotanical investigations conducted across 14 Balkan nations, 1289 plant taxa were recorded, guiding the selection of specific species for deeper exploration. The project consortium compiled these findings into a structured report using standard ethnobotanical indices—Relative Frequency of Citations, Use Value, and Fidelity Level. As a result, 240 plant species emerged as promising candidates for potential medicinal uses. Subsequent fieldwork yielded over 170 plant samples gathered from their native habitats, thereby fulfilling Milestone MS1, which stipulated the collection of at least 100 specimens. These samples have been deposited in the Laboratory of Pharmacognosy and Natural Products Chemistry (LPNPC) herbarium, and their distribution patterns throughout the Balkan Peninsula are currently under review.

In parallel, a range of *in silico* models targeting specific biological pathways were established. The results of these models showcase the successful integration of docking calculations and molecular dynamics (MD) simulations through the automated Enalos Asclepios KNIME platform, providing an end-to-end solution, encompassing protein and ligand preparation, docking, MD system setup, and trajectory analysis. Its streamlined workflow ensures consistency and reproducibility, which are critical for large-scale computational research.

Protein and Ligand Preparation involved meticulous refinement to model receptor-ligand interactions accurately. Protein receptors were processed for homology modelling to address structural gaps, remove heteroatoms, and adjust ions and hydrogens at physiological pH. The refined structures were converted into docking-compatible formats with force fields such as ff14SB for proteins and GAFF2 for ligands ensuring reliable parameterization. Ligands then underwent 3D structure minimization and energy optimization. Charge assignments along with geometry optimization, further prepared the ligands for docking and MD simulations. Ligands with promising docking scores underwent Molecular Mechanics Generalized Born Surface Area (MM-GBSA) rescoring to evaluate binding affinities in greater detail. This multi-tiered approach enabled the prioritization of ligands with optimal interaction profiles.

The study targeted six enzymes associated with skin disorders: elastase, hyaluronidase, tyrosinase, collagenase, cycloxygenase and xanthine oxidase. A virtual screening of approximately 210,000 compounds identified top-ranking ligands through iterative rescoring. From this pool, 200 ligands per target underwent MM-GBSA analysis, leading to the identification of promising candidates: 1ELE-VS_7 for elastase, 1FCV-VS_10 for hyaluronidase, 2Y9X-VS_15 for tyrosinase, and 3NVY-VS_17 for xanthine oxidase. Then, bioinformatics analysis further detailed the binding site characteristics and interactions. For elastase, stabilization was achieved through a catalytic triad (Ser, His, Asp) and hydrophobic residues (Phe223, Arg226, Val224). Hyaluronidase displayed a polar-rich binding groove (∼30 Å × 10 Å) mediated by Asp111, Glu113, and Tyr227. Tyrosinase interacted near its binuclear copper site, with hydrogen bonding involving Arg268, Arg321, and Asn260. Xanthine oxidase featured a cavity adjacent to the quercetin binding site, accommodating bulkier ligands and stabilizing them through residues like Gln1194, Gln1040, and Ser1080. Ligand-receptor complex stabilization involved hydrogen bonds and van der Waals forces. Elastase formed intricate hydrogen bond networks complemented by pi-pi stacking. Hyaluronidase enhanced ligand stability through backbone hydrogen bonds from Ser303 and Ser304. Tyrosinase demonstrated robust interaction networks with both polar and hydrophobic residues, while xanthine oxidase relied on hydrophobic interactions, notably involving Phe798 and Met1038.

### 3.2 Phytochemical investigations

**Fig. 3:**
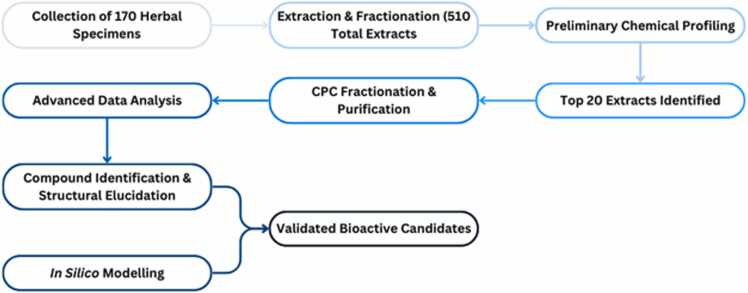
Flowchart illustrating the integrated extraction, chemical profiling, fractionation, and in silico modeling approach employed to identify and characterize bioactive plant compounds for therapeutic applications.

During the phytochemical investigations performed in the project (Fig. 3), significant progress was made regarding the identification and characterization of bioactive compounds derived from diverse plant species. The collected 170 herbal specimens were extracted using environmentally friendly technologies, adhering to protocols inspired by the traditional use of plant materials and at least three extracts were prepared for each herbal sample, resulting in 510 plant extracts aiming to recover the largest proportion of bioactive ingredients and mimic traditional recipes. Leveraging state-of-the-art analytical techniques, the project identified over 500 compounds from 16 plant species, showcasing the richness of bioactive constituents within carefully selected plant extracts. The application of methodologies such as Centrifugal Partition Chromatography (CPC), Ultra-High-Performance Liquid Chromatography coupled with High-Resolution Mass Spectrometry (UHPLC-HRMS), and Nuclear Magnetic Resonance (NMR) spectroscopy proved to be crucial for the successful isolation and characterisation of these compounds. Additionally, 30 secondary metabolites, including unique flavonoids, phenolic acids, and triterpenoids, were successfully isolated and structurally elucidated, leading to the creation of a valuable repository of compounds with significant therapeutic potential.

Moreover, in order to identify preparations with remarkable biological properties against enzymes associated with skin diseases, the chemical content of the extracts was initially evaluated using Total Phenolic Content (TPC), Total Flavonoid Content (TFC) and High-Performance Thin Layer Chromatography (HPTLC) analysis. Having performed this chemical profiling, the 20 most promising extracts were revealed, and they were then subsequently subjected to qualitative characterisation using Liquid Chromatography-Mass Spectrometry (LC-MS). These extracts were then fractionated using CPC, and the fractions were chemically characterised and assessed for biological activity.

So as to further streamline the process of compound identification and evaluation, advanced analytical techniques were integrated. More specifically, dereplication strategies effectively eliminated redundant isolation of known compounds, enabling a more efficient discovery of novel bioactive agents while UHPLC-HRMS facilitated detailed chemical profiling. Moreover, advanced multivariate data analysis and heterocovariance methods extracted meaningful chemical patterns from complex datasets. These approaches allowed for accurate identification of bioactive compounds and their correlation with observed biological activities, enhancing the project’s precision and efficacy. The multivariate data analysis and heterocovariance approach were particularly valuable in analysing spectroscopic and chromatographic data, allowing the identification of active components within the fractions and directing them to further biological evaluation. Additionally, these data were cross-referenced with commercial databases to enhance compound validation.

Through the above analysis, key plant species, including *Agrimonia eupatoria*, *Cistus creticus*, *Hypericum empetrifolium*, and *Centaurea salonitana*, were identified as promising candidates due to their rich bioactive profiles and historical ethnopharmacological relevance as their extracts exhibited high concentrations of phenolics, flavonoids, and triterpenoids - known for their therapeutic properties. Now, regarding their therapeutic applications, the identified bioactive compounds displayed noteworthy properties, including significant tyrosinase and collagenase inhibitory activities, underscoring their potential in anti-aging and whitening products. Furthermore, compounds such as momordicosides, catechins, and myricetin derivatives exhibited wound-healing and antioxidant properties, supporting tissue regeneration and combating oxidative stress.

**Fig. 4:**
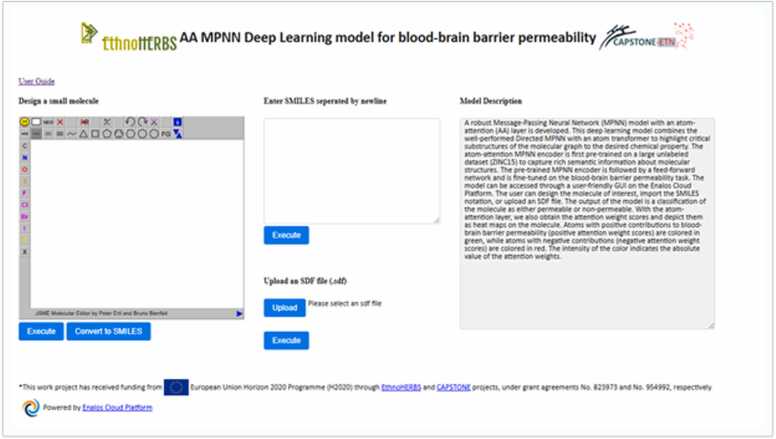
Web Application of the MPNN Deep learning model hosted in the Enalos Cloud Platfrom, developed during the EthnoHERBS Project.

In parallel, predictive modeling techniques for molecular properties, such as permeability, were utilized to conduct *in silico* activity and safety assessments of isolated compounds. Advanced methodologies, including atom-attention MPNNs and contrastive learning, were applied to predict key properties such as molecular permeability, potential toxicity, and interactions with biological targets. Web applications for the models used were developed and are freely available online on the Enalos Cloud Platform ( Fig. 4). These approaches provided a robust framework for prioritizing compounds with favorable pharmacologiscal profiles, enabling more efficient and targeted selection of candidates for subsequent experimental analysis.

### 3.3 Biological evaluation

**Fig. 5:**
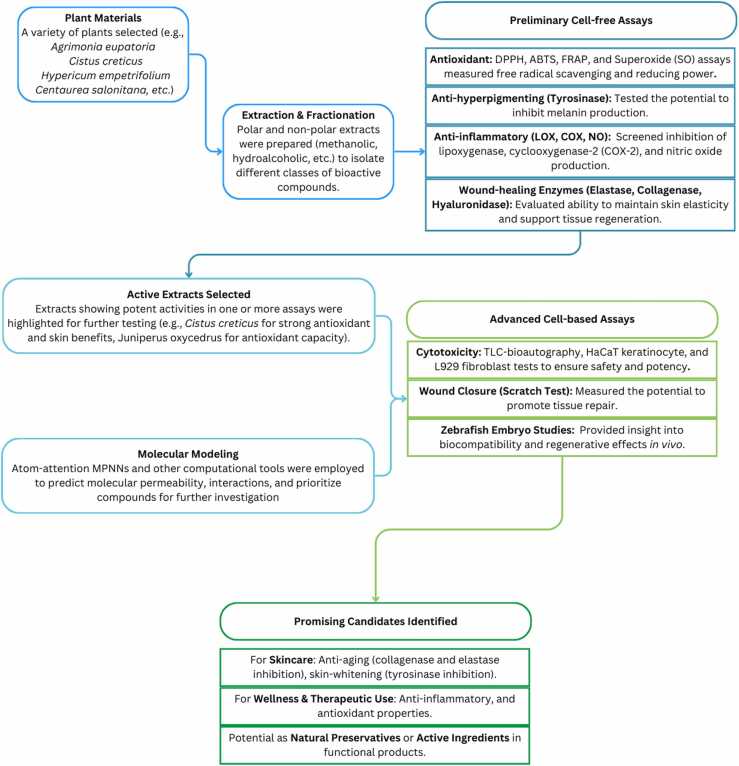
Schematic overview of the EthnoHERBS project workflow for biological evaluation, from plant extraction and bioactivity screening (antioxidant, anti-inflammatory, etc.) to advanced in vitro and in vivo testing, molecular modeling, and final selection of promising plant-derived extracts for skincare and wellness applications.

Throughout the EthnoHERBS project, notable strides were made in assessing the pharmacological effects of plant-derived extracts through a blend of cell-free and cell-based methods (an overview is presented in Fig. 5). Antioxidant potential was systematically measured using DPPH (2,2-diphenyl-1-picrylhydrazyl), ABTS (2,2’-azino-bis(3-ethylbenzothiazoline-6-sulphonic acid)), FRAP (Ferric Reducing Antioxidant Power), and SO (Superoxide Anion) assays. The DPPH test evaluated the extracts’ free radical-scavenging capability, revealing that polar formulations (e.g., methanolic and hydroalcoholic) showed stronger effects compared to non-polar ones. In the ABTS assay, powerful antiradical activity emerged from *Anchusa undulata, Ononis viscosa* and H*ypericum empetrifolium* (EC50 values of 15 mg/mL, 39 mg/mL and 85 mg/mL, respectively). The FRAP test highlighted elevated reducing capacities in *Cistus creticus* subsp. *eriocephalus* and *Sedum sediforme*, while the SO assay indicated more than 90 % inhibition of superoxide radicals by various polar plant extracts.

Beyond antioxidant effects, a variety of cell-free assays were used to examine the extracts, fractions, and isolated substances for anti-melanoma capacity (via tyrosinase assay), wound-healing attributes (targeting collagenase), and anti-inflammatory effectiveness (lipoxygenase and cyclooxygenase assays, NO and SO scavenging activity).

It is noteworthy that the extracts of *Cistus creticus subsp. eriocephalus and Origanum dictamnus* showed the highest anti-collagenase activity followed by *Rhus coriaria, Cistus salvifolius, Cistus creticus subsp. creticus* and subsp. eriocephalus as well as *Cotinus coggygria* showing inhibitions bigger than 90 %. Moreover, *Cistus creticus subsp. creticus* methanolic extract exhibited a concentration-dependent inhibitory activity of elastase enzyme, highlighting its potential use in anti-aging skin care.

*Sedum sediforme, Cistus creticus subsp. creticus*, *Centaurea raphanina*, *Centaurea salonitana* and *Hypericum empetrifolium* displayed potent COX-2 inhibition at 250 mg/mL while *Cistus creticus subsp. creticus*, *Sedum sediforme, Plumbago europaea* and *Thymbra capitata* showed the highest inhibition of COX-1 activity at 100 μg/mL concentration. Among the plant extracts tested for lipoxygenase inhibition, the most active (showing IC50 values below 100 μg/mL) were the methanolic extracts of *Thymbra capitata* and *Sedum sediforme* (IC50: 28.0 and 39.4 μg/mL respectively). Meanwhile, tyrosinase inhibition assays pointed to significant activity in flavonoid-rich extracts, with the strongest anti-tyrosinase activity observed by *Morus alba* followed by *Cistus creticus subsp. eriocephalus, Cotinus coggygria, Cistus creticus subsp. Criticus* and *Pinus nigra*. Tests on wound healing and cytotoxicity further confirmed the therapeutic value of these extracts. TLC-bioautography methods were used to screen cytotoxicity, and highly active samples underwent evaluation through cell-based assays and *in vivo* models. Investigations using HaCaT keratinocytes and L929 fibroblasts revealed that the extract of Cistus creticus subsp. creticus significantly boosted cell viability and promoted wound closure. Scratch assays confirmed an expedited healing process, and zebrafish embryo studies demonstrated the safety of this extract and its role in supporting tissue regeneration. Iron-chelating assays confirmed the extracts’ ability to lessen oxidative damage linked to metal ions. Particularly noteworthy were *Ononis viscosa* (95.26 % Fe^2 +^ chelation at 400 µg/mL), *Momordica charantia* (94.04 %), and *Anchusa undulata* (83.45 %).

### 3.4 Conservation and development

The key objectives of WP4 include conserving and propagating a selection of plant species identified in other WPs, producing superior plantlets via asexual propagation under strict organic guidelines, and incorporating these species into organic cropping systems. Additionally, the WP focuses on refining large-scale cultivation protocols for organic agriculture, applying environmentally friendly approaches for pilot-scale production of principal natural products, and conducting both formulation and stability analyses to finalize innovative product lines. Thus far, transplant materials have been gathered from wild habitats following stringent collection procedures, resulting in over 20 chosen species. From these, 9 plantlets have undergone successful *in vitro* asexual propagation to retain desirable traits.

## 4 Discussion

EthnoHERBS, to the best of our knowledge is the first systematic multidisciplinary attempt to record and evaluate information on SE European traditional knowledge, to use cutting edge chemistry technologies, to evaluate in a high-throughput manner the high biodiversity of SE European flora to discover and develop innovative ‘cosmeceutical’ products against skin disorders. As a project, it showcases how cutting-edge extraction procedures, thorough structural analysis, and pharmacological assessments can be effectively combined to utilize bioactive compounds obtained from ethnopharmacological data. By blending traditional herbal knowledge with advanced scientific techniques, the EthnoHERBS project creates a link between historical ethnopharmacological wisdom and modern healthcare solutions. This integration not only helps preserve biodiversity and cultural traditions but also confirms the industrial relevance of these bioactive compounds. In particular, extracts with notable phenolic and flavonoid content exhibited remarkable antioxidant activity, verified through DPPH and FRAP assays. These outcomes emphasize the extracts’ suitability in skincare, as they address aging, support skin whitening, and counteract oxidative stress. In terms of future therapeutic possibilities, this project highlights applications across skincare, pharmaceuticals, and nutraceuticals. Validated extracts pave the way for anti-aging products through collagenase and elastase inhibition, while tyrosinase-blocking agents tackle hyperpigmentation. Additionally, these extracts show promise in the nutraceutical field by offering functional health benefits that bolster overall well-being.

Via the *in silico* methodologies utilised through the project, the integration of advanced computational methods and ethnobotanical knowledge for efficient identification of bioactive compounds with therapeutic potential was highlighted. With MM-GBSA calculations and MD simulations accurate re-scoring of ligand binding affinities and detailed insights into protein-ligand stability were provided, streamlining the identification of promising ligands for cosmeceutical applications, targeting enzymes linked to skin health, including elastase, tyrosinase, hyaluronidase, and xanthine oxidase. Moreover, ethnobotanical insights guided the computational screening of plant-derived secondary metabolites, bridging traditional knowledge with modern drug discovery. The Enalos Asclepios KNIME Workflow facilitated a transparent and reproducible computational pipeline, with binding site visualisations and ligand tables detailing predicted ΔGbinding values and chemical structures. The promising plants identified through *in vitro* biological evaluation and chemical profiling, were selected for propagation material production. They were cultivated to retain their desirable traits, and their extracts were prepared at a pilot scale and optimized for industrial implementation. Pilot-scale batches of natural products were developed into innovative cosmeceutical products addressing consumer needs for skin disorder treatments.

Beyond its purely scientific results, the EthnoHERBS research project generated significant economic and social benefits at the individual, organizational, and European levels, enhancing the strategic competitiveness of both academic and non-academic partners, particularly SMEs in Europe. By integrating entrepreneurial skills with scientific expertise, the project improved career prospects for researchers, increased international and interdisciplinary mobility, and fostered collaboration between sectors. It drove higher impact research and innovation, contributing to Europe's knowledge-based economy, boosted R&I capacity, and created new business opportunities. The project strengthened networks, improved interactions between public and private sectors, and supported the development of innovative solutions based on plant knowledge.

## 5 Dissemination and communication

The EthnoHERBS project has placed a strong emphasis on dissemination and communication to maximise its visibility, share its results with a broad audience, and foster engagement with both the scientific community and the public. A comprehensive dissemination strategy was implemented, supported by a publicly accessible website, https://www.EthnoHERBS.eu/, which serves as a central communication tool. The website provides detailed information about the project’s objectives, progress, and outcomes, ensuring transparency and accessibility for all stakeholders.

One of the project’s key achievements in dissemination is the publication of over 25 peer-reviewed articles in high-impact scientific journals. These publications reflect the rigorous scientific efforts of the consortium and significantly contribute to the academic discourse surrounding ethnopharmacology, natural product research, and sustainable biodiversity use. In addition to scientific publications, the project’s goals and findings were promoted on scientific platforms, beneficiaries' websites, and through newsletters and dissemination articles published in both scientific and non-scientific magazines. A notable recognition of the project's impact came through a dedicated feature in the **HORIZON EU Research and Innovation Magazine**, which highlighted EthnoHERBS’ role in preserving Europe’s medicinal plants from extinction. The article, available here, underscores the significance of integrating traditional herbal knowledge with cutting-edge scientific approaches to ensure biodiversity conservation and sustainable healthcare solutions.

Workshops played a pivotal role in the project’s dissemination efforts, facilitating direct engagement with the research community and stakeholders. The 1st Workshop provided an initial platform to present the project's objectives and early findings. The 2nd Workshop, titled “Natural Products Research,” took place during the 2nd Panhellenic Congress of Ethnopharmacology in 2024 in Ioannina, Greece. This event attracted over 100 participants, including academics, industry representatives, and students, showcasing the project’s significant progress in natural product research. A 3rd Workshop titled “Innovation in cosmeceutical and nutraceutical industry” is scheduled for March 2025, during the final meeting of the project, reflecting the project’s outcomes, engaging with the broader scientific community and maintaining momentum in knowledge dissemination. In addition to workshops, a Mid-Term Meeting (MTM) was held as an online one-day event and two webinars were organized by TecMinho regarding Technology Transfer and Entrepreneurship as well as by Apivita on Cosmeceutical and EU Compliance.

## 6 Conclusion

The EthnoHERBS project exemplifies the successful integration of traditional herbal knowledge with advanced scientific methodologies to address pressing global challenges in biodiversity conservation and innovative healthcare solutions. By systematically exploring the rich ethnobotanical heritage of the Balkans, the project has not only uncovered a wealth of bioactive compounds with therapeutic potential but also established sustainable practices for their cultivation and application. Key achievements include the identification and characterization of novel bioactive compounds, advancements in environmentally friendly extraction techniques, and the validation of therapeutic properties through comprehensive biological evaluations. The project’s interdisciplinary approach has enabled the development of cosmeceutical products targeting skin disorders, highlighting their relevance in anti-aging, wound healing, and anti-inflammatory applications. These outcomes underscore the potential for innovation within the pharmaceutical, nutraceutical, and cosmetic industries. The project’s collaborative framework, involving academic and non-academic partners across multiple regions, has fostered knowledge exchange and set a benchmark for international research initiatives. Through its robust dissemination efforts and eco-friendly methodologies, EthnoHERBS has demonstrated a model for sustainable resource utilization that benefits both local communities and global markets. Moving forward, the project's findings pave the way for further research and development, bridging the gap between traditional practices and modern scientific advancements to achieve long-term societal and environmental impact.

## CRediT authorship contribution statement

**Afantitis Antreas:** Formal analysis, Resources, Software, Supervision, Writing – review & editing. **Kasiotis Konstantinos:** Data curation, Formal analysis, Methodology. **Aligiannis Nektarios:** Conceptualization, Data curation, Funding acquisition, Project administration, Supervision, Visualization, Writing – review & editing. **Gardikis Konstantinos:** Data curation, Formal analysis, Investigation, Methodology. **Dias Paula:** Funding acquisition, Project administration, Software. **Oluški Mirko:** Investigation, Methodology, Resources. **Dajic Stevanovic Zora:** Data curation, Formal analysis, Investigation, Methodology, Project administration. **Billia Anna Rita:** Data curation, Formal analysis, Investigation, Methodology, Resources. **Zivkovic Jelena:** Data curation, Formal analysis, Investigation, Methodology, Resources. **Petrangolini Giovanna:** Data curation, Formal analysis, Investigation, Methodology. **Dias Alberto:** Data curation, Formal analysis, Investigation, Methodology. **Zouraris Dimitrios:** Methodology, Software, Writing – original draft. **Vasileiou Panagiotis:** Data curation, Resources, Software, Writing – original draft. **Graikou Konstantia:** Formal analysis, Project administration, Writing – original draft, Writing – review & editing, Data curation. **Dimitrov Vladimir:** Data curation, Formal analysis, Investigation, Resources. **Ramón Muñoz Montaño Juan:** Data curation, Formal analysis, Investigation, Methodology. **Hristova Hristina:** Investigation, Methodology, Resources. **Iliev Hristo:** Data curation, Formal analysis, Investigation, Methodology.

## Declaration of Competing Interest

The authors declare that there is no conflict of interest regarding the publication of this paper.
